# The Assignment of the Absolute Configuration of Non-Cyclic Sesquiterpenes by Vibrational and Electronic Circular Dichroism: The Example of *Chiliadenus lopadusanus* Metabolites

**DOI:** 10.3390/biom11121902

**Published:** 2021-12-18

**Authors:** Giuseppe Mazzeo, Alessio Cimmino, Giovanna Longhi, Marco Masi, Antonio Evidente, Sergio Abbate

**Affiliations:** 1 Dipartimento di Medicina Molecolare e Traslazionale, Università degli Studi di Brescia, Viale Europa 11, 25123 Brescia, Italy; giuseppe.mazzeo@unibs.it (G.M.); giovanna.longhi@unibs.it (G.L.); 2 Dipartimento di Scienze Chimiche, Università di Napoli Federico II, Complesso Universitario Monte Sant’Angelo, 80126 Napoli, Italy; alessio.cimmino@unina.it (A.C.); marco.masi@unina.it (M.M.); antonio.evidente@unina.it (A.E.)

**Keywords:** *Chiliadenus lopadusanus*, farnesane-type sesquiterpenes, 9-hydroxynerolidol, 9-oxonerolidol and chiliadenol B, VCD, ECD, absolute configuration, DFT calculations

## Abstract

9-Hydroxynerolidol, 9-oxonerolidol, and chiliadenol B are three farnesane-type sesquiterpenoids isolated from *Chiliadenus lopadusanus* that have shown an interesting activity against human pathogens as Gram+ and Gram− bacteria resistant to antibiotics. However, the absolute configuration (AC) of these interesting sesquiterpenes has not been assigned so far. Vibrational and electronic circular dichroism spectra have been recorded and correlations are pointed out for the three compounds. Density functional theory (DFT) calculations are used in conjunction with Mosher’s method of investigation to assign AC. Statistical analysis is considered to quantitatively define the choice of AC from VCD spectra.

## 1. Introduction

The bioactive metabolites produced by plants, microorganisms, lichens, and algae had shown to possess a plethora of different biological activities such as phytotoxic, antibacterial, antiviral, anticancer, antitumor, algicide, antifungal, enzyme inhibiting, immunostimulant, antiplatelet aggregation, cytotoxic and antiplasmodial activities. They belong to all different classes of natural compounds as alkaloids, hydrocarbons, lipids, sterols, esters, fatty acids, derivatives of amino acids, terpenoids and aromatic compounds, etc., [1,2,3,4]. Among them, many could have potential practical application in agriculture as natural safe pesticides [5,6] and others could be the active principle of new drugs [7,8,9].

Natural products, in particular, those with a new carbon skeleton, could represent an efficient solution to discover and develop new drugs to overcome antimicrobial resistance and treat biofilm-related infections. This is a real emergency since in recent decades many pathogens causing human infection rapidly increased their antibiotic resistance reducing the efficacy of therapies. Recently the antibiotic activity of natural compounds, such as *epi*-epoformin, sphaeropsidone, and sphaeropsidin A was tested against some species of Gram+ and Gram− bacteria which are considered common opportunistic pathogens inducing human heavy infections. Their combination increased their efficacy while sphaeropsidin A also inhibited biofilm formation. These results are very promising and suggested to some of us how to develop a suitable drug formulation, in particular, for wound protection against serious infection advancement [10].

These results prompted further research on fungi and plants to identify the bioactive natural substances with antibiotic activity against human pathogens as antibiotic-resistant bacteria. Thus, a screening of endemic plants collected in different regions of the Mediterranean basin was carried out to identify the best plant to reach this objective. The organic extract of *C. lopadusanus*, among all collected plants, showed high inhibition of some pathogenic bacteria growth. *C. lopadusanus* Brullo is an endemic plant growing spontaneously on Lampedusa Island, the largest Island of the Pelage archipelago, about 100 km from the North Africa coast and 200 km from Sicily coast [11].

**Figure 1:**
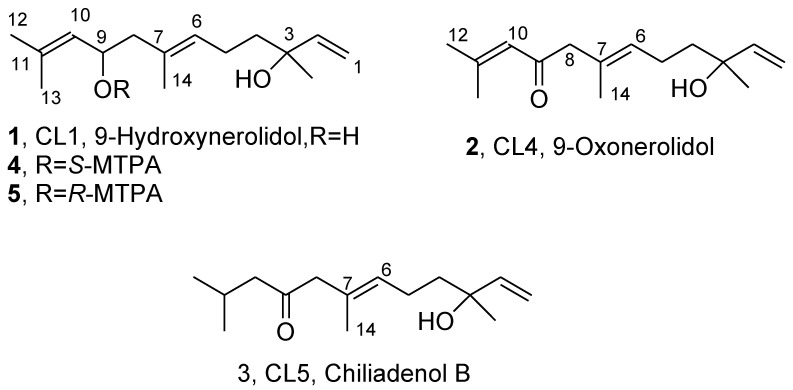
Structures of 9-hydroxynerolidol, 9-oxonerolidol, and chiliadenol B (**1**–**3**).

The organic extract of *C. lopadusanus* showed very strong activity against both Gram+ and Gram− bacteria pathogens for the human species, which showed antibiotic resistance. This activity was essentially related to the presence of three farnesane-type sesquiterpenes, namely 9-hydroxynerolidol, 9-oxonerolidol, and chiliadenol B (**1**–**3**, Figure 1), respectively. The sesquiterpenes **1**–**3** were identified by comparing their optical rotation data (specific optical rotation: [α]^25^_D_ = +5.2 for **1**, +14.4 for **2**, and +4.0 for **3**) and spectroscopic data (IR, UV, 1D ^1^H and ^13^C NMR and ESIMS) with the literature [12]. Their purity >98% was ascertained by ^1^H NMR, ESI MS, and HPLC.

9-Hydroxynerolidol and 9-oxonerolidol (**1** and **2**) were isolated for the first time together from *Solanum melongena* [13]. The absolute configuration (AC) of 1 was tentatively assigned thereby reducing 9-oxonerolidol (**2**), and comparing its ^13^C NMR data with the natural one. According to Stoessl et al. [13], the product of the reduction was the expected mixture of two diastereomers, while the natural compound **1** appeared to be a mixture (~4:1) of two diastereomers and both natural and synthetic diastereomeric mixtures of **1** have no optical activity. The AC of the bioactive form of sesquiterpene **2** was determined based on ^1^H NMR and ORD data [13,14,15]. The AC of chiliadenol B (**3**) up to now has not been determined [16].

Considering these results that practically do not permit the unambiguous AC assignment of **1** and **2** and the still missing assignment of AC of **3** and considering strictly relationships between the AC and the biological activity of naturally occurring substances [17,18], we concluded that different methods, i.e., chiroptical ones, are needed to assign the AC of the three farnesane-type sesquiterpenes **1**–**3**. Indeed, the latter papers demonstrate that the determination of AC is not a mere exercise to complete the knowledge about the chemistry of natural products but may tell us about their way of functioning as pharmaceutical agents.

This Manuscript describes the AC assignment of the three compounds, comprised of the configurations of the stereogenic carbon atoms C-9 for **1** and C-3 for all three sesquiterpenes, using the advanced Mosher method and extensive use of vibrational circular dichroism (VCD) spectroscopy. Indeed, while the former approach has met with numerous achievements and has been critically reviewed by Cimmino et al. [19], the latter technique has been increasingly used through the years [20,21]. For natural products, it has been used with success [22,23,24,25]. Even more relevant for the present work are the VCD and IR studies by Merten et al. [26,27] on linear diterpene natural products extracted from *Bifurcaria Bifurcata* algae. Herein we will make use of VCD and IR absorption data in the mid-IR region, and of IR spectra in the CH and OH stretching regions, the latter spectroscopic data still being relevant in the discrimination of diastereomers, albeit they not being chiroptical data. VCD in the CH and OH-stretching regions will be cursorily considered (signals there are not fully reliable) and some limited use will be made of the corresponding IR spectra. Furthermore, following the recommendation of Polavarapu et al. [28], we will consider electronic circular dichroism (ECD) data in conjunction with VCD: we think that the synergistic use of the two types of data is particularly opportune in the present case since the expected number of possible conformers makes this study difficult, already noticed by Merten et al., for some diterpenes, namely elegandiol and other linear diterpenes from *Bifurcaria* family [26,27].

## 2. Materials and Methods

### 2.1. General Experimental Procedures

Optical rotation was measured in CHCl_3_ solution by a P-1010 digital polarimeter (Jasco, Tokyo, Japan). ^1^H and ^13^C NMR spectra were recorded at 400 and 100 MHz in CDCl_3_ by Bruker spectrometers (Karlsruhe, Germany). ESIMS were recorded using LC/MS ESIMS-TOF system (Agilent 6230B, HPLC 1260 Infinity) (Milan, Italy). The HPLC separation was performed using a Phenomenex LUNA (C18 (2) 5u 150 mm × 4.6 mm) (Torrance, CA, USA). Analytical, preparative, and reverse-phase TLCs were carried out on silica gel (Merck, Kieselgel 60, F254, 0.25, 0.5 mm, and RP-18 F254s respectively) plates (Merck, Darmstadt, Germany). The spots were visualized by exposure to UV radiation, or by spraying first with 10% H_2_SO_4_ in MeOH, and then with 5% phosphomolybdic acid in EtOH, followed by heating at 110 °C for 10 min. Column chromatography was performed using silica gel (Kieselgel 60, 0.063–0.200 mm) (Merck, Darmstadt, Germany).

### 2.2. Plant Material

Whole aerial parts of *C. lapodusanus* plants were collected fresh in Lampedusa Island (Italy) by Mr. Fabio Giovanetti and identified by Prof. G. Surico, University of Florence, Italy. The plant specimen is deposited in the collection of the Department of Plant Pathology, University of Florence. The air-dried sample was obtained as recently reported [10].

### 2.3. Isolation of Fungal Metabolites and Synthesis of Ancillary Products

Plant material (450 g) was extracted by H_2_O/MeOH (1/1, *v/v*) and the organic extract was purified by a combination of column and TLC as recently reported [12] to obtain all samples as pure oils, namely for 9-hydroxynerolidol (**1**, 12.4 mg), 9-oxonerolidol (**2**, 11.7 mg) and chiliadenol B (**3**, 20.4 mg).

#### 2.3.1. Hemisynthesis of **4**

*9-O-(S)-α-Methoxy-α-trifluoromethyl-α-phenylacetate (MTPA) ester of* **1** (**4**). To **1** (1.0 mg) in pyridine (100 μL), (R)-(−)-MPTA-Cl (10 μL) was added. The mixture was carried out for 4 h at 25 °C and quenched by adding methanol and toluene. The mixture was evaporated using an N_2_ stream. The residue (1.3 mg) was purified by TLC, eluted with CHCl_3_/isoPrOH (95/5, *v/v*), yielding **4** as a homogeneous oil (0.8 mg). It had: ^1^H NMR, see Table 1; ESI MS (+) spectrum, m/z 455 [M + H]^+^.

#### 2.3.2. Hemisynthesis of **5**

*9-O-(R)-α-Methoxy-α-trifluoromethyl-α-phenylacetate (MTPA) ester of* **1** (**5**): To **1** (1.0 mg) in pyridine (100 μL) was added (S)-(+)-MPTA-Cl (10 μL) and the reaction was performed as above reported. The crude residue (1.2 mg) was purified by TLC eluted with CHCl_3_/isoPrOH (95/5, *v/v*), affording **5** as a homogeneous oil (0.7 mg). 5 had: ^1^H NMR, see Table 1; ESI MS (+) spectrum, m/z 455 [M + H]^+^.

### 2.4. VCD and ECD Spectroscopies

VCD experimental studies were conducted using a Jasco FVS 6000 FTIR instrument equipped with a ZnSe photo-elastic modulator (PEM), working at 50 kHz modulation, placed past a wire grid linear polarizer and with lock-in amplifier after detection, with either an MCT or an InSb liquid-N_2_ cooling device for the regions 850–1800 cm^−1^ and 2500–4000 cm^−1^ respectively. For the latter region, we report only the IR spectra, since experimental VCD spectra did not exhibit a good enough signal-to-noise ratio. Samples were dissolved in CCl_4_ with the following concentrations: 0.02 M in 5 mm Infrasil quartz cuvette for the OH-stretching region, 0.05 M in 1 mm Infrasil quartz cuvette for the CH-stretching region, and 0.25 M in 200 μm BaF_2_ cell for the mid-IR region. 6000 scans were acquired for each case, and similar spectra were taken for the solvent and subtracted out. ECD experimental studies were conducted with the use of a Jasco 815SE instrument with samples dissolved in acetonitrile at 0.001 M concentration in 1 mm quartz cuvettes. 5 scans per spectra were acquired using 1 mm Suprasil quartz cuvettes. ECD spectra of the solvent were recorded in the same conditions and subtracted thereafter from the sample ECD spectra. UV spectra were obtained from the same apparatus, from voltage-adjusted DC signals.

### 2.5. Calculations: From MM to DFT

The Gaussian16 package [29] was extensively used to theoretically investigate compounds **1**, and, to a lesser extent, **2**. We decided to work for **1** with the (3*S*,9*S*) and (3*R*,9*S*) stereoisomers. We first used the MM routine to retain all conformers within the energy interval 0–5 kcal/mol; we then undertook the quantum DFT method to define conformers within 0–2 kcal/mol (B3LYP/6-311++G(2d,p) level of theory). Finally, for the defined conformers we calculated VCD spectra using the Stephens theory [30] embedded in Gaussian16 for the calculation of rotational strengths, assigning Lorentzian band-shapes to each fundamental transition (10 cm^−1^ bandwidth) and averaging them with Boltzmann population factors based on ΔG. No anharmonic correction was tried, as illustrated by us earlier on simpler cases [31,32]. Also due to missing treatment of anharmonicity, we do not report the comparison of computed and calculated spectra for the CH/OH stretching regions, which notoriously are most affected by that phenomenon [31,32]. VCD spectra for the (3*R*,9*R*) and (3*S*,9*R*) stereoisomers were generated by simply reversing the signs of spectra calculated for the (3*S*,9*S*) and (3*R*,9*S*) stereoisomers. The two sets of IR spectra are coincident with the original (3*S*,9*S*) and (3*R*,9*S*) choices. Other levels of theory, namely B3LYP/TZVP, were tried but found unsatisfactory. ECD spectra were calculated for the very same conformers using the TD/CAM-B3LYP functional and 60 states; Gaussian band shapes were employed with 0.2 eV bandwidth.

## 3. Results and Discussion

The organic extract of dried leaves of *C. lopadusanus* was purified as reported in the Materials and Methods section to afford the three farnesane type 9-hydroxynerolidol, 9-oxonerolidol, and chiliadenol B (**1**–**3**) as pure oils. Their purity >98% was ascertained by ^1^H NMR, ESIMS, and HPLC analyses.

Preliminarily, the relative stereochemistry of **1**–**3** and, in particular, that of a double bond between C-6 and C-7 was assigned by NOESY experiments**.** In the NOESY spectrum of **1**, **2,** and **3** the lack of correlation between Me-14 with H-6 in all compounds confirmed the E configuration assigned in literature [13,19] to the double bond between C-6 and C-7.

At that point, the AC at C-9 of **1** and C-3 of all the three compounds remains to be determined.

**Figure 2:**
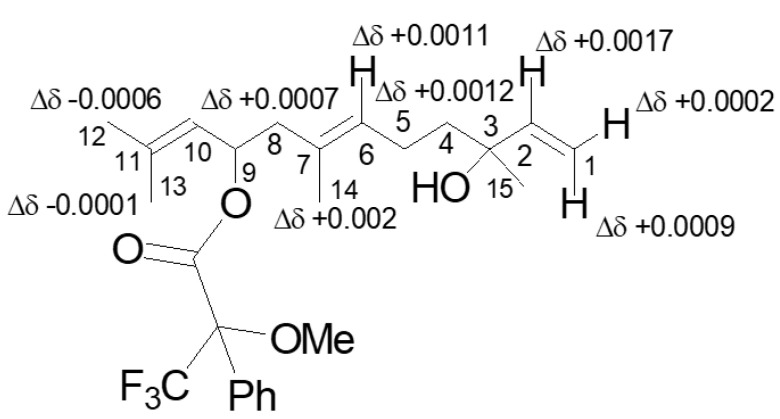
Structures of 9-*O*-*S*- and 9-*O*-*R*-MTPA esters of 9-hydroxynerolidol esters (**4** and **5**, respectively), reporting the Δδ value of each proton system.

A first assay to determine AC at C-9 of **1** consisted in the application of the advanced Mosher’s method. Sesquiterpene **1** was reacted with (R)-(−)- and (S)-(+)-MTPACl and yielded the corresponding monoesters **4** and **5** and their ^1^H NMR spectra were carefully recorded and the values for the chemical shifts are reported in Table 1. Subtracting the chemical shifts of **5** from those of **4** (Table 1), the Δδ (**4**–**5**) values for almost all the protons were determined and described pictorially in Figure 2.

Applying model A as reported in Cimmino et al. [19] the (*S*) configuration was assigned at C-9.

**Figure 3:**
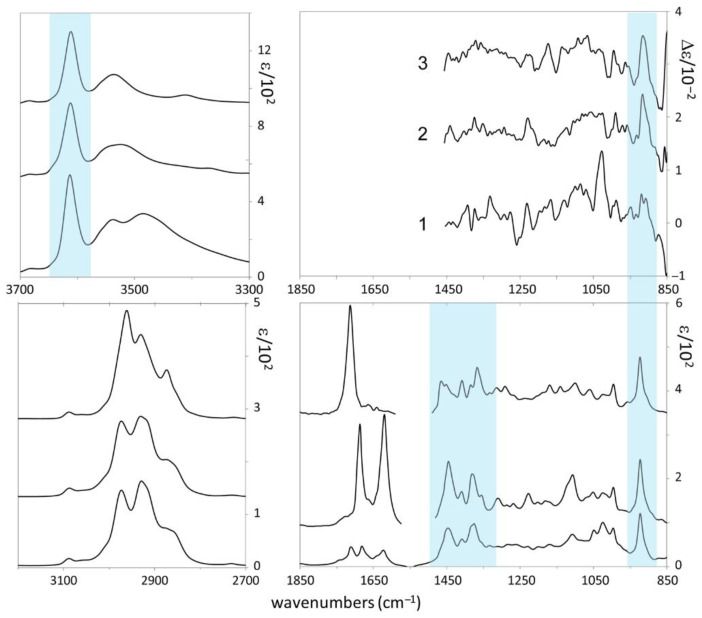
**Right:** Experimental VCD (top) and IR absorption (lower) spectra of 9-hydroxynerolidol, R=H (**1**), of 9-oxonerolidol (**2**), and chiliadenol (**3**) in the mid-IR. **Left**: Experimental IR absorption spectra of 9-hydroxynerolidol, R=H (**1**), of 9-oxonerolidol (**2**), and chiliadenol (**3**) in the OH-stretching (top) and CH-stretching (lower) regions. Features common to the three molecules are evidenced.

**Figure 4:**
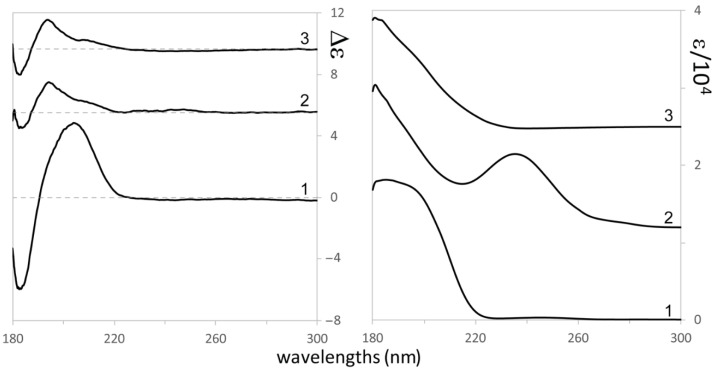
Experimental ECD (left) and UV absorption (right) spectra of 9-hydroxynerolidol (**1**), of 9-oxonerolidol (**2**), and chiliadenol (**3**).

In Figure 3 we report the experimental IR and VCD spectra of the three compounds and in Figure 4 the corresponding UV and ECD spectra. We first seek common features in the spectra of the three molecules, namely we adopt an empirical correlative approach, and then we move to the comparison of experimental with DFT calculated spectra. We do this, since, due to the high number of conformers (*vide infra*), the exact determination of AC may be out of reach by the standard method. A few facts are worth pointing out from IR and VCD in the mid-IR region:

(i) At low wavenumbers, a feature at 921 cm^−1^ stands out, which exhibits negative VCD in all three cases and strong IR absorption. We believe this feature to be important for AC determination of stereo-carbon 3, common to all three molecules, possessing the same configuration there;
(ii) at 1383 and 1450 cm^−1^ there is an IR doublet for all three compounds, which had been noticed also in refs [25,26]; no large VCD signal corresponds to these characteristic IR features;
(iii) the VCD spectrum of **1** is generally more intense than those for **2** and **3** and has some similarities with three out of four linear diterpenes’ VCD spectra by Merten et al. [26,27], the most notable being the strong positive VCD band at ca. 1030 cm^−1^_;_
(iv) in the C=C/C=O stretching regions a weak IR triplet is recorded for **1**, (1630, 1683, and 1716 cm^−1^) much in the same way as observed by Merten et al. [26,27]. The three features are due to C=C stretchings, which are known to exhibit weak absorption [33]. Interestingly with just one C=O in both compounds, the spectral behavior in **2** and **3** is different: in **2** two strong bands appear at ca. 1630 and 1683 cm^−1^, due to the coupled C=C/C=O stretchings; in **3** the single strong band at 1716 cm^−1^ is visible and is due to the isolated C=O stretching, far away from all C=C moieties.

In the higher wavenumber CH- and OH-stretching regions we point out the overall similarity in the three compounds of the CH stretching IR absorption spectrum. Several VCD spectra of cyclic terpenes [34,35,36] were recorded and presented in the past in the CH-stretching region, with similar IR spectra as found here: we mention them here for sake of completeness, but we will not discuss them, for reasons reported in the Materials and Methods section. The IR absorption spectra in the OH-stretching region looks more interesting since it exhibits a sharp feature at 3612 cm^−1^, common to all three compounds, and a broad feature at 3542 cm^−1^ in all three compounds, plus another broad hump centered at 3487 cm^−1^ for **1**. Following Paoloni et al. [32] (see also the more recent work by Hartwig and Suhm [37]), who carefully investigated the cases of diols, we think that the 3612 cm^−1^ is due to the OH-stretching in either the 3- or 9- position acting as an acceptor of H-bond and behaving almost as an OH-stretching “free” from H-bond. We believe the 3542 cm^−1^ feature instead to be due to OH stretching in donor intramolecular H-bonding for the 3 position (common to **1**, **2,** and **3**) and the 3487 cm^−1^ feature to be due to OH stretching in donor intramolecular H-bonding for the 9 position (present only in **1**).

Let us now come to consider Figure 4. A bisignate ECD feature extending from ca. 215 to 180 nm stands out in the spectrum and one might be tempted to correlate the three spectra. Yet some differences are noticed, which are important to consider: in **1** the intensity of the couplet is much more intense (from 5 to 10 times) than in **2** and **3**, which are quite similar among themselves. Besides, the wavelengths of the observed features show differences, which are worth noticing: the positive feature in **1** is centered at 205 nm, with a shoulder at 193 nm; the negative feature is at 182 nm. In **2** and **3** instead, while the negative component is at 182 nm, the positive component is structured with a shoulder at 213 nm and the main positive band at 193 nm. Earlier work on the diene chromophore [38,39] reviewed by Lightner and Gurst [40], address the possibility that the diene chromophore is distorted, so as to form a dissymmetric chromophore, or instead of being a symmetric chromophore dissymmetrically perturbed by the nearby oxygen atom. In the following, we will fully rely on TD-DFT calculations, which generally supersede previous interpretations [41]. Besides, the strong UV absorption at ca. 240 nm for **2**, corresponding to a weak positive ECD effect, calls for a *n*→π* transition, strongly coupled to diene transitions, in a way recalling what happens in the 1600–1700 cm^−1^ region of the IR absorption spectrum of **2**. The coupling may also justify enhanced UV absorption [40].

**Figure 5:**
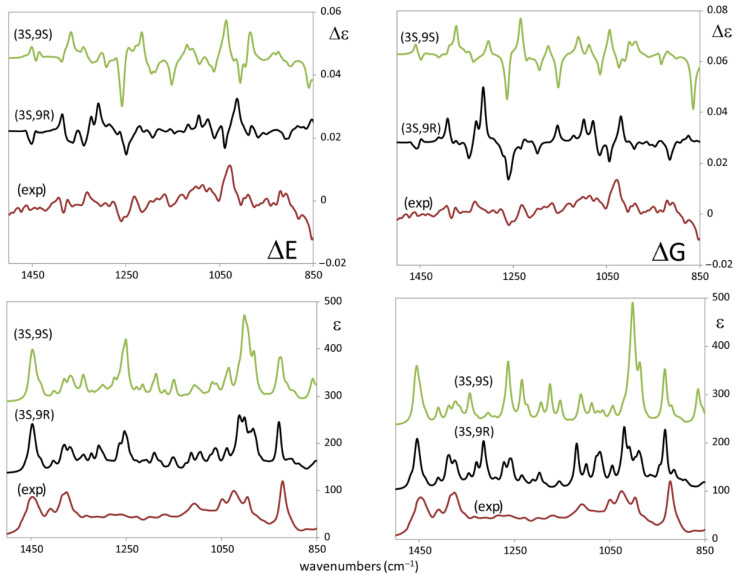
Comparison of experimental VCD (top) and IR absorption (bottom) spectra of 9-hydroxynerolidol, R=H (**1**), with corresponding DFT-calculated spectra of (3*S*,9*R*)-**1** and (3*S*,9*S*)-**1** with two different Boltzmann averages: on the left average carried out based on ΔE, on the right average carried out based on ΔG. Scaling factors 0.975 and 0.97 for (3*S*,9*R*)-**1** and (3*S*,9*S*)-**1** respectively. Scaling Factor for (3*S*,9*S*) = 0.965; SF for (3*S*,9*R*) = 0.975.

After the above empirical analysis of our chiroptical experiments for **1** (for which measured [α]_D_^25^ = +4), for **2** ([α]_D_^25^ = +14), and for **3** ([α]_D_^25^ = +5), all OR measurements being carried out on chloroform diluted solutions, let us compare the DFT calculated IR and VCD spectra for **1** to corresponding experimental ones in the mid-IR region. Results are shown in Figure 5 for two choices of AC (3*S*,9*S*)-**1** and (3*S*,9*R*)-**1**. The calculated results for the other two possible configurations, namely (3*R*,9*R*)-**1** and (3*R*,9*S*)-**1**, are not presented since their VCD can be obtained from those for (3*S*,9*S*)-**1** and (3*S*,9*R*)-**1** by simply reversing the signs, while their IR is the same as for (3*S*,9*S*)-**1** and (3*S*,9*R*)-**1**. We limited calculations to **1**, since the number of predicted conformers is huge, namely 15 for (3*S*,9*S*)-**1** and 30 for (3*S*,9*R*)-**1** (with ca. 400 conformers admitted by Molecular Mechanics): in the former cases, the predicted 15 conformers have ΔE energies above the global minimum conformation ≤3 kcal/mol, in the latter case the 30 predicted conformers have ΔE ≥ 2.5 kcal/mol above the global minimum. For reasons which will become clear in the subsequent discussion we averaged the calculated spectra for separate conformers through Boltzmann factors proportional to either e^−(^^ΔE/RT)^ or e^−(^^ΔG/RT)^: results, though not drastically dissimilar, present some differences, which makes one choice preferable over the other. Upon inspection and based on IR spectra (IR is not a chiroptical technique, but is able to distinguish between diastereomers), we think that the best choice is (3*S*,9*R*) over (3*S*,9*S*); we also think that averaging over ΔE provides better fitting than averaging over ΔG. Figure 3 shows that IR spectra in the OH stretching region indicate the presence of several intramolecular HB, thus favoring compact conformers, as predicted for the most populated conformers according to the ΔE statistics (*vide infra*). To a lesser extent than for IR, the same choice of AC and of average also holds for VCD.

The choice made by visual inspection is quantitatively confirmed by calculating the similarity indices, as is commonly done in natural products chiroptical spectroscopy [42,43,44,45,46]. Indeed, as shown in the Supplementary Material (Figure S1 and Table S1) for the VCD spectra: for (3*S*,9*R*) we have 0.4 for parameter S.I. (ΔG) and 0.55 (ΔE), while for (3*S*,9*S*) we have 0.25 for parameter S.I. (ΔG) and 0.3 (ΔE). Similar conclusions are arrived at for parameter Sim_NN. In the Supplementary Material file, the results for other choices of the scaling factors around the values presented in the figures are explored, but the response is unambiguous, even though the differences between the parameters’ values are not that big. (For the correct use of scaling factors, please consult NIST at: CCCBDB listing of precalculated vibrational scaling factors. Most importantly, based on this method, we also conclude that the (3*R*,9*R*) and (3*R*,9*S*) AC are to be excluded a fortiori; while we have to admit that the difference between the calculated indices for (3*S*,9*S*) and for (3*S*,9*R*) AC is not that great, in the remaining cases (3*R*,9*R*) and (3*R*,9*S*) we have even negative similarity SI indices, a safe argument not to accept the latter choices for AC.

Coming now to a more detailed comment on the results, we invite the interested reader to consult the Supplementary Material file, where the energetics of the conformers and the 3D-representation of the main conformers are given (Table S2 for the energetics in ΔE and ΔG for AC (3*S*,9*R*) and Figures S2–S4 for the 3D-representation of (3*S*,9*R*)-conformers ordered in ΔE-population factors; Figures S5–S7 for 3D-representation of (3*S*,9*R*)-conformers ordered in ΔG-population factors; Table S3 for the energetics in ΔE and ΔG for AC (3*S*,9*S*) and Figures S2–S4 for the 3D-representation of (3*S*,9*S*)-conformers ordered in ΔE-population factors; Figures S5–S7 for 3D-representation of (3*S*,9*S*)-conformers ordered in ΔG-population factors). We observe that for (3*S*,9*R*) one has 3 conformers above 10% population-based on ΔE-statistics, 2 conformers above 10% based on ΔG-statistics; 6 conformers between 10% and 5% population-based on ΔE, 1 conformer in the same range based on ΔG. For (3*S*,9*S*) one has 3 conformers above 10% population-based on ΔE, 1 conformer above 10% based on ΔG; 2 conformers between 10% and 5% population-based on ΔE, 2 conformers in the same range based on ΔG. A sizeable number of conformers is found to have population factors smaller than 5%. It is interesting to note that most of the preferred conformers from ΔE appear kind-of-rolled up and compact due to the presence of intramolecular hydrogen bonds (HB) between the two OH groups, while the preferred ΔG-conformers are more elongated, with conformers exhibiting HB having smaller population factors. We believe that in the latter case, the absence of HB assures a higher larger entropic contribution, thus minimizing ΔG. This provides us with a further criterion to prefer the ΔE-choice over the ΔG-choice, since from the IR spectra in the OH stretching region (Figure 3) we may appreciate that internal HB is quite present.

**Figure 6:**
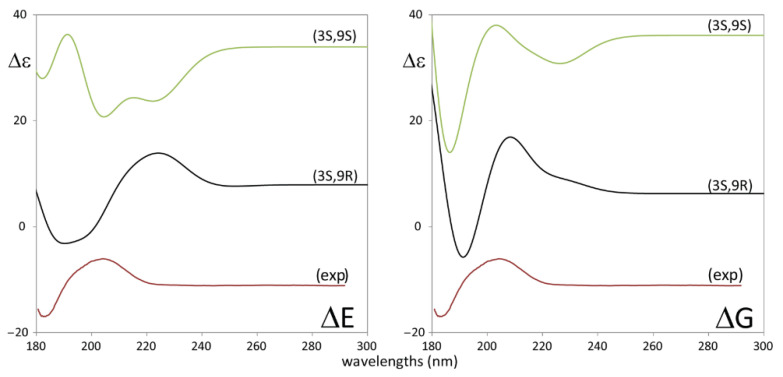
Comparison of experimental ECD (top) spectra of 9-hydroxynerolidol, (**1**), with corresponding TD-DFT calculated spectra of (3*S*,9*R*)-**1** and (3*S*,9*S*)-**1** with two different Boltzmann averages: on the left average carried out based on ΔE, on the right average carried out based on ΔG. No wavelength shift applied to calculated spectra. No shift was applied.

Coming finally to consider the ECD spectra, in Figure 6 we report, in strict analogy to what done for VCD in Figure 5, the comparison of calculated spectra for (3*S*,9*S*) and (3*S*,9*R*) choices with the experimental ECD spectra. We think that, while based on ΔG-averages, there is no preference of (3*S*,9*S*) over (3*S*,9*R*) (or just a slight preference of the latter over the former, due to the presence of a non-observed negative hump at a low wavelength in the calculated ECD spectrum of the former, based on ΔE we should reject the (3*S*,9*S*)-choice.

As a final comment on this “Results and Discussion” section, we notice that there is a contradiction between the VCD/ECD/DFT results and the Mosher’s method NMR data about the configuration of stereogenic carbon 9 of sesquiterpene **1**. The former methods indicate a (slight) preference for (*R*), the latter one for (*S*). This is a bit of a problem and in the conclusions section, we’ll list the facts in favor of either choice. Instead, the choice for stereogenic carbon atom 3 is definitely (*S*), within the limits of the difficulty of the present problem, related to the huge number of conformers and to interactions with the solvent, which have not been treated in the present work (related to the latter aspect, we point out that VCD spectra were recorded in CCl_4_, an apolar, very symmetric solvent). Stereogenic carbon 3 is shared by all three compounds examined here and it is reassuring to state that, due to spectroscopic similarities, especially in the ECD spectra, the configuration of carbon 3 is the same in the three compounds and is (*S*), a characteristic which appears robust and maintained in the members of the whole family. Moreover, we are glad to be in accord with the configuration of carbon 3 with the previous conclusions by Merten et al. (2017) on linear diterpenes extracted from algae [26,27].

## 4. Conclusions

In this work, we have investigated, by chiroptical spectroscopic methods, namely VCD and ECD allied to DFT calculations and ancillary theoretical-statistical analysis, the three related linear farnesane-type sesquiterpenes 9-hydroxynerolidol (**1**), 9-oxonerolidol (**2**), and chiliadenol B (**3**), isolated from *C. lopadusanus*, which is an endemic and native plant growing in the Lampedusa Island (Sicily, Italy). The three compounds have shown interesting activity against human pathogens, e.g., Gram+ and Gram− bacteria resistant to the antibiotic. We believe the AC assignment is beneficial to understand the action mechanism of potential drugs that could be based on these compounds. We have established, through VCD and ECD spectra allied with DFT calculations that the configuration of the stereogenic carbon atom 3 is (*S*). For compound **1** VCD and ECD show a slight preference for stereogenic carbon 9 to be in the (*R*) configuration (vis-à-vis the similarity index parameters), while the application of the advanced Mosher method, which has recently found widespread use on natural products [18], indicates that the (*S*) choice for the configuration of carbon atom 9 is preferable. This contradictory result should be viewed with some benevolence since the number of admissible conformers is between 20 and 50, which makes the problem quite challenging for any technique.

Finally, we remark that the very same AC assignment for carbon 3 was made also for some linear diterpenes extracted from algae of the Mediterranean Sea, a fact which may not be so fortuitous, shedding some light onto the biochemistry of the two different organisms, one terrestrial and one marine, yet from the same geographic area, from which the different linear terpenes were extracted.

## Figures and Tables

**Table 1:** ^1^H NMR data of 9-*O*-(*S*)- and 9-*O*-(*R*)-MTPA of 9-hydroxynerolidol esters (**4** and **5**, respectively) ^1^.

	4	5
position	δ_H_ (*J* in Hz)	δ_H_ (*J* in Hz)
1	5.2148 br d (17.5) 5.0649 dd (10.8, 1.2)	5.2150 (1H) br d (17.5) 5.0658 dd (10.7, 1.1)
2	5.9134 dd (17.7, 10.8)	5.9151 dd (17.5, 10.7)
5	2.0454 m (2H)	2.0466 m (2H)
6	5.1582 br t (6.9)	5.1593 br t (6.7)
H-8A	2.6624 d (5.5)	2.6631 d (5.6)
Me-12 ^2^	1.3150 s	1.3144 s
Me-13 ^2^	1.2822 s	1.2821 s
Me-14	1.5778 s	1.5798 s
OMe	4.6763 s	4.6766 s
Ph	7.5175–7.3555 (5H) m	7.584–7.3524 (5H) m

^1^ The chemical shifts are in δ values (ppm) from TMS. ^2^ These two signals could be exchanged.

## Supplementary Materials

The following are available online at https://www.mdpi.com/article/10.3390/biom11121902/s1, Figure S1: graphical representations of SI as a function of scaling factors SF for the AC two choices (3*S*,9*R*) and (3*S*,9*S*); Figures S2–S4 for the 3D-representation of (3*S*,9*R*)-conformers ordered in ΔE-population factors; Figures S5–S7 for 3D-representation of (3*S*,9*R*)-conformers ordered in ΔG-population factors; Figures S8–S11: the 3D-representation of (3*S*,9*S*)-conformers ordered in ΔE-population factors. Figures S12–S14: 3D-representation of (3*S*,9*S*)-conformers ordered in ΔG-population factors, Table S1: Similarity Indices SI and Sim_NN for selected values of scaling factors for the four possible AC choices, (3*S*,9*R*), (3*S*,9*S*), (3*S*,9*R*), (3*S*,9*R*); Table S2: energetics in ΔE and ΔG for AC (3*S*,9*R*); Table S3: energetics in ΔE and ΔG for AC (3*S*,9*S*).
