# Supplementary material for: The Assignment of the Absolute Configuration of Non-Cyclic Sesquiterpenes by Vibrational and Electronic Circular Dichroism: The Example of Chiliadenus lopadusanus Metabolites
Source: Biomolecules. 2021 Dec 18;11(12):1902. doi: 10.3390/biom11121902 (PMC8699476; doi:10.3390/biom11121902)
Supplement: Supplementary file 1 [file biomolecules-11-01902-s001.zip › biomolecules-1500174-supplementary.pdf]

The assignment of the absolute configuration of non-cyclic sesquiterpenes by vibrational and electronic circular dichroism: the example of chiliadenus lopadusanus metabolites

Giuseppe Mazzeo , Alessio Cimmino, Giovanna Longhi, Marco Masi, Antonio Evidente, and Sergio Abbate

LIST OF FIGURES AND TABLES

Table S1: Similarity Indices SI and Sim_NN for selected values of scaling factors for the four possible AC choices, (3S,9*R*), (3S,9*S*), (3R,9S), (3R,9R).
Figure S1: graphical representations of SI as function of scaling factors SF for the AC two choices (3S,9*R*) and (3S,9*S*).
Table S2: energetics in ΔE and ΔG for AC (3S,9R).
Figures S2-S4 for the 3D-representation of (3S,9R)-conformers ordered in ΔE-population factors;
Figures S5-S7 for 3D-representation of (3S,9R)-conformers ordered in ΔG-population factors.
Table S3: energetics in ΔE and ΔG for AC (3S,9S).
Figures S8-S11: the 3D-representation of (3S,9S)-conformers ordered in ΔE-population factors.
Figures S12-S14: 3D-representation of (3S,9S)-conformers ordered in ΔG-population factors.

TABLE S1

ΔG spectra average

Isomer	S.I.(1.00)	S.I.(0.99)	S.I.(0.98)	S.I.(0.97)	S.I.(0.96)	S.I.(0.95)
(3S,9R)	0.10	0.31	0.42	0.27	0.12	0.08
(3S,9S)	0.03	-0.06	-0.04	0.24	0.19	-0.02
**Isomer**	**Sim_NN(1.00)**	**Sim_NN(0.99)**	**Sim_NN(0.98)**	**Sim_NN(0.97)**	**Sim_NN(0.96)**	**Sim_NN(0.95)**
(3S,9R)	0.05	0.18	0.26	0.15	0.07	0.04
(3S,9S)	0.01	-0.03	-0.02	0.13	0.10	-0.01

ΔE spectra average

Isomer	S.I.(1.00)	S.I.(0.99)	S.I.(0.98)	S.I.(0.97)	S.I.(0.96)	S.I.(0.95)
(3S,9R)	0.22	0.38	0.54	0.29	0.08	0.10
(3S,9S)	-0.04	-0.10	0.04	0.26	0.28	0.09
**Isomer**	**Sim_NN(1.00)**	**Sim_NN(0.99)**	**Sim_NN(0.98)**	**Sim_NN(0.97)**	**Sim_NN(0.96)**	**Sim_NN(0.95)**
(3S,9R)	0.12	0.22	0.34	0.16	0.04	0.05
(3S,9S)	-0.04	-0.05	0.02	0.15	0.16	0.05

ΔG spectra average

Isomer	S.I.(1.00)	S.I.(0.99)	S.I.(0.98)	S.I.(0.97)	S.I.(0.96)	S.I.(0.95)
(3R,9S)	-0.10	-0.31	-0.42	-0.27	-0.12	-0.01
(3R,9R)	-0.03	0.06	0.04	-0.24	-0.19	0.02
**Isomer**	**Sim_NN(1.00)**	**Sim_NN(0.99)**	**Sim_NN(0.98)**	**Sim_NN(0.97)**	**Sim_NN(0.96)**	**Sim_NN(0.95)**
(3R,9S)	-0.05	-0.18	-0.26	-0.15	-0.07	-0.03
(3R,9R)	-0.01	0.03	0.02	-0.13	-0.10	0.01

ΔE spectra average

Isomer	S.I.(1.00)	S.I.(0.99)	S.I.(0.98)	S.I.(0.97)	S.I.(0.96)	S.I.(0.95)
(3R,9S)	-0.22	-0.43	-0.54	-0.29	-0.08	-0.15
(3R,9R)	0.08	0.10	-0.04	-0.26	-0.28	-0.09
**Isomer**	**Sim_NN(1.00)**	**Sim_NN(0.99)**	**Sim_NN(0.98)**	**Sim_NN(0.97)**	**Sim_NN(0.96)**	**Sim_NN(0.95)**
(3R,9S)	-0.12	-0.25	-0.34	-0.16	-0.04	-0.08
(3R,9R)	0.04	0.05	-0.02	-0.15	-0.16	-0.05

FIGURE S1

ΔG spectra average

Isomer	S.I.(1.00)	S.I.(0.99)	S.I.(0.98)	S.I.(0.97)	S.I.(0.96)	S.I.(0.95)
(3S,9R)	0.10	0.31	0.42	0.27	0.12	0.08
(3S,9S)	0.03	-0.06	-0.04	0.24	0.19	-0.02
**Isomer**	**Sim_NN(1.00)**	**Sim_NN(0.99)**	**Sim_NN(0.98)**	**Sim_NN(0.97)**	**Sim_NN(0.96)**	**Sim_NN(0.95)**
(3S,9R)	0.05	0.18	0.26	0.15	0.07	0.04
(3S,9S)	0.01	-0.03	-0.02	0.13	0.10	-0.01

ΔE spectra average

Isomer	S.I.(1.00)	S.I.(0.99)	S.I.(0.98)	S.I.(0.97)	S.I.(0.96)	S.I.(0.95)
(3S,9R)	0.22	0.38	0.54	0.29	0.08	0.10
(3S,9S)	-0.04	-0.10	0.04	0.26	0.28	0.09
**Isomer**	**Sim_NN(1.00)**	**Sim_NN(0.99)**	**Sim_NN(0.98)**	**Sim_NN(0.97)**	**Sim_NN(0.96)**	**Sim_NN(0.95)**
(3S,9R)	0.12	0.22	0.34	0.16	0.04	0.05
(3S,9S)	-0.04	-0.05	0.02	0.15	0.16	0.05

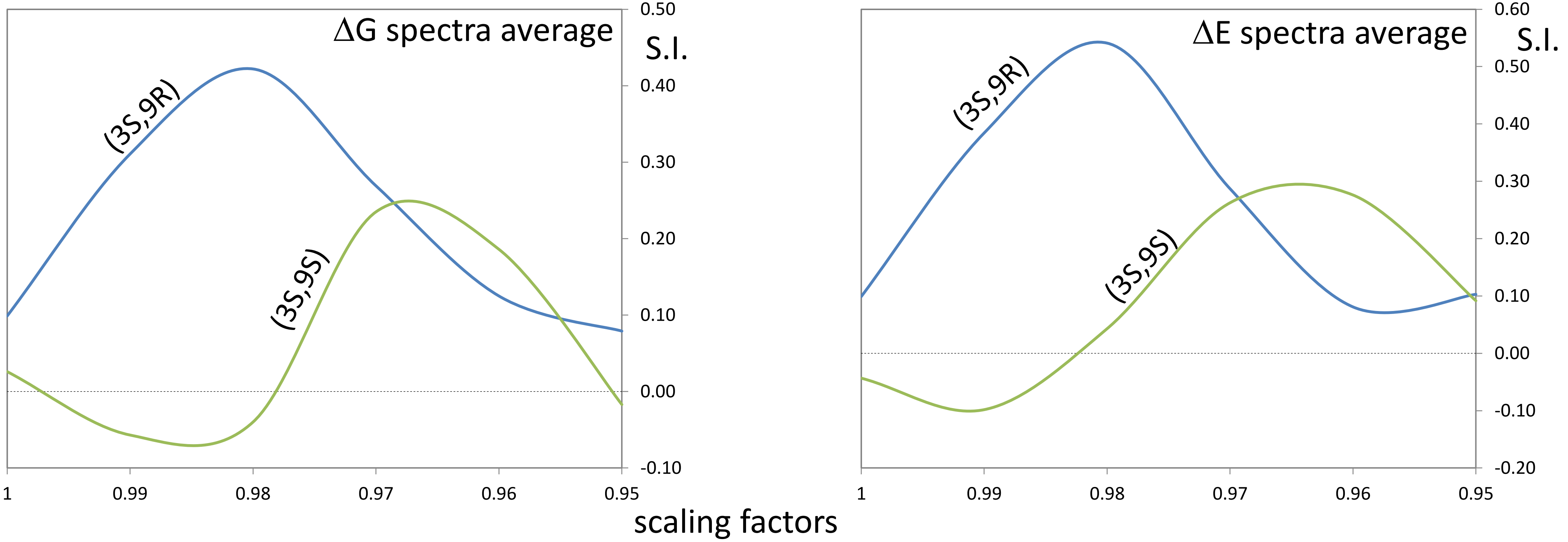

TABLE S2

1: AC (3*S*,9*R*) Computed Poputation Factors

ΔE

CONFORMERS	ΔE	% pop
337	0.000000	12.5
44	0.042573	11.7
360	0.134554	10.0
23	0.307920	7.5
21	0.307982	7.5
26	0.328297	7.2
6	0.329300	7.2
79	0.329551	7.2
58	0.486803	5.5
18	0.559409	4.9
165	0.947522	2.5
124	0.981757	2.4
323	1.104899	1.9
196	1.230111	1.6
118	1.334256	1.3
250	1.403289	1.2
60	1.432820	1.1
19	1.568879	0.9
81	1.596844	0.8
227	1.663368	0.8
59	1.681802	0.7
262	1.879683	0.5
104	1.909842	0.5
269	1.967150	0.5
80	1.975928	0.4
154	1.978248	0.4
65	1.978310	0.4
2	2.169545	0.3
28	2.188669	0.3
127	2.400971	0.2

Conformational Search B3LYP/6-31G* on 368 conformers.
Re-optimized with frequency calculated at B3LYP/6-311++G(2d,p)

$ Original numbering of the conformers from the MM conformational search has been maintained

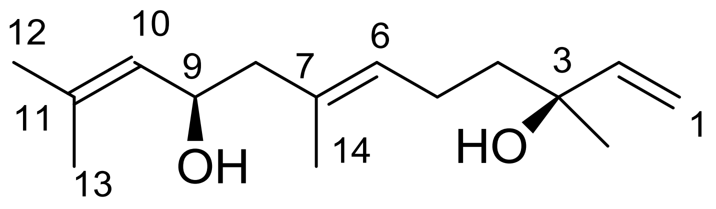

1, CL1, 9-Hydroxynerolidol

ΔG

CONFORMERS	ΔG	% pop
337	0.000000	28.1
360	0.030723	26.7
323	0.779361	7.5
104	1.010097	5.1
80	1.065273	4.7
79	1.106655	4.3
23	1.136124	4.1
6	1.350558	2.9
44	1.586937	1.9
18	1.622049	1.8
227	1.636470	1.8
59	1.673463	1.7
269	1.701051	1.6
58	1.771902	1.4
26	1.826451	1.3
165	2.101077	0.8
124	2.129292	0.8
21	2.215191	0.7
118	2.361909	0.5
60	2.415204	0.5
196	2.597034	0.4
28	2.650956	0.3
81	2.855358	0.2
262	2.980758	0.2
65	2.982012	0.2
154	2.985147	0.2
250	3.069792	0.2
19	3.377022	0.1
2	3.546312	0.1
127	3.853542	0.0

(3*S*,9*R*)-**1**

FIGURE S2

Populations; Conformers for (≥10%)-P.F.-based on ΔE ΔE

CONFORMERS	ΔE	% pop
337	0.000000	12.5
44	0.042573	11.7
360	0.134554	10.0
23	0.307920	7.5
21	0.307982	7.5
26	0.328297	7.2
6	0.329300	7.2
79	0.329551	7.2
58	0.486803	5.5
18	0.559409	4.9
165	0.947522	2.5
124	0.981757	2.4
323	1.104899	1.9
196	1.230111	1.6
118	1.334256	1.3
250	1.403289	1.2
60	1.432820	1.1
19	1.568879	0.9
81	1.596844	0.8
227	1.663368	0.8
59	1.681802	0.7
262	1.879683	0.5
104	1.909842	0.5
269	1.967150	0.5
80	1.975928	0.4
154	1.978248	0.4
65	1.978310	0.4
2	2.169545	0.3
28	2.188669	0.3
127	2.400971	0.2

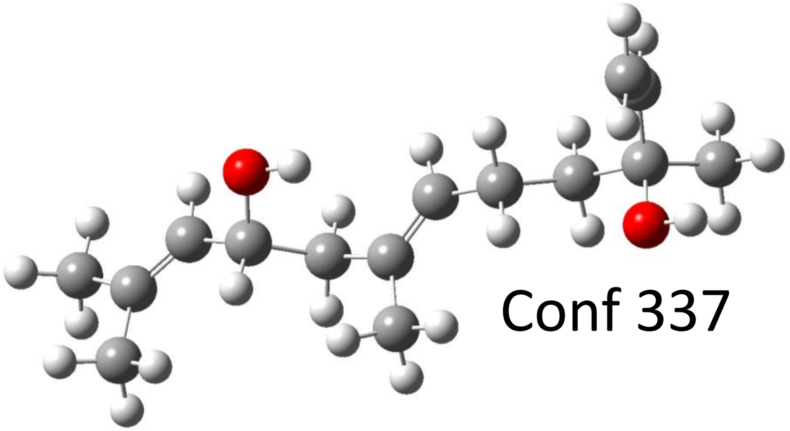
Conf 337

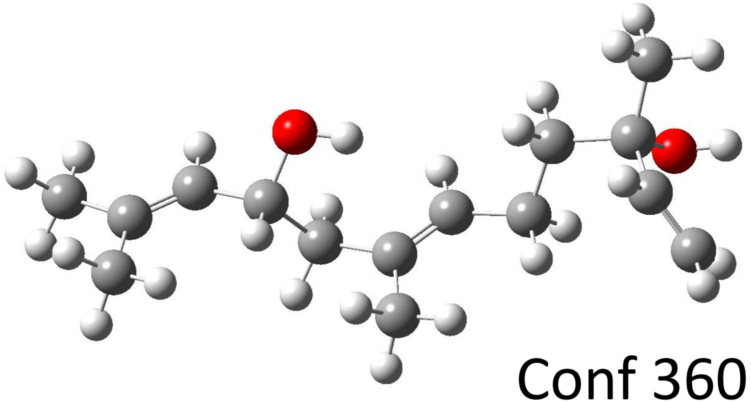
Conf 360

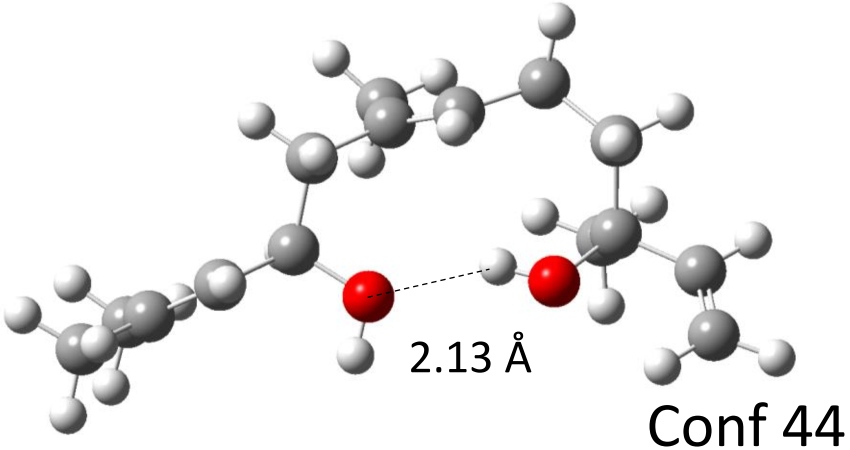

Conf 44

(3*S*,9*R*)-**1**

FIGURE S3

Populations; Conformers for P.F. (9%-5%) based on ΔE

ΔE

CONFORMERS	ΔE	% pop
337	0.000000	12.5
44	0.042573	11.7
360	0.134554	10.0
23	0.307920	7.5
21	0.307982	7.5
26	0.328297	7.2
6	0.329300	7.2
79	0.329551	7.2
58	0.486803	5.5
18	0.559409	4.9
165	0.947522	2.5
124	0.981757	2.4
323	1.104899	1.9
196	1.230111	1.6
118	1.334256	1.3
250	1.403289	1.2
60	1.432820	1.1
19	1.568879	0.9
81	1.596844	0.8
227	1.663368	0.8
59	1.681802	0.7
262	1.879683	0.5
104	1.909842	0.5
269	1.967150	0.5
80	1.975928	0.4
154	1.978248	0.4
65	1.978310	0.4
2	2.169545	0.3
28	2.188669	0.3
127	2.400971	0.2

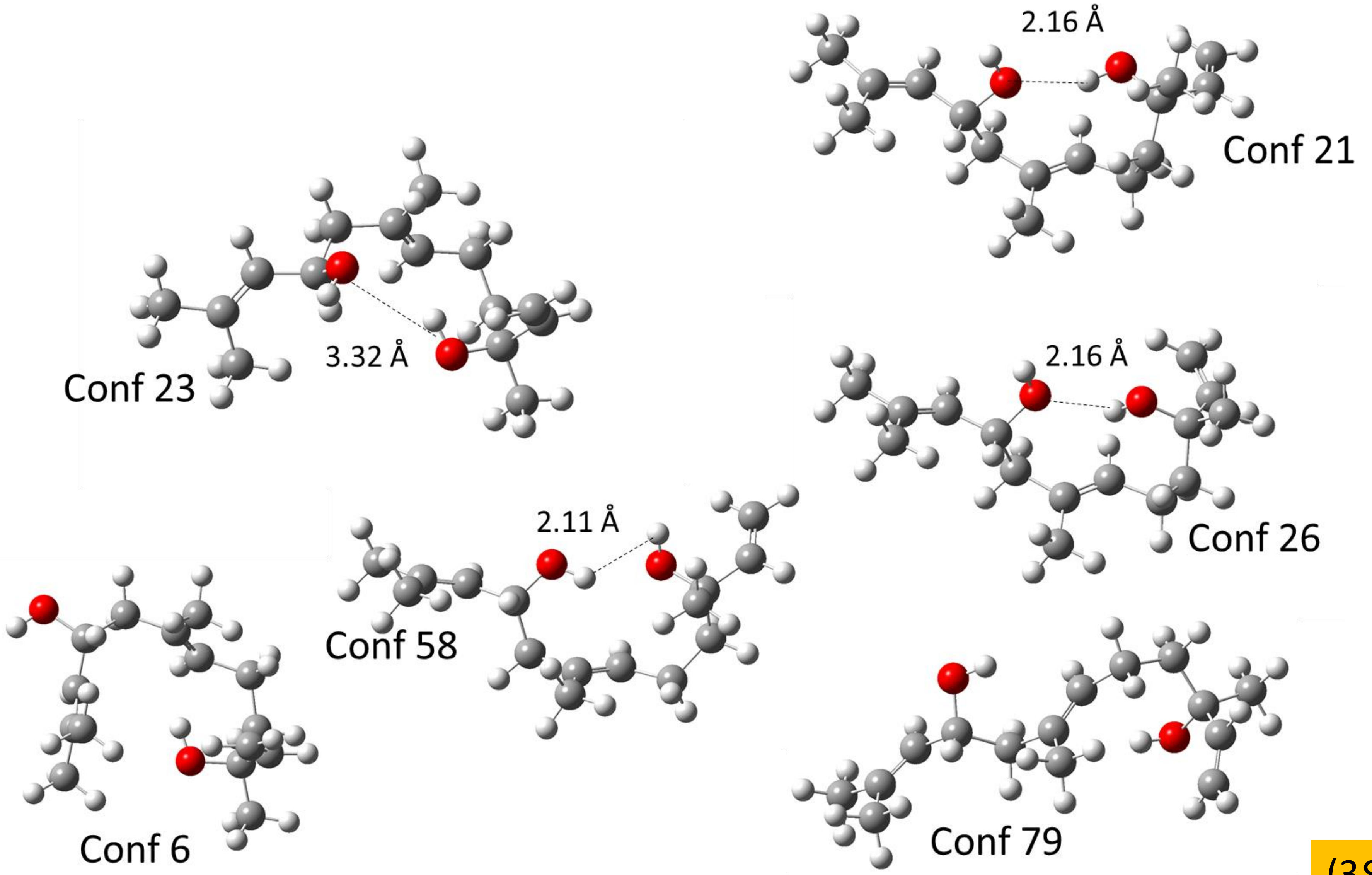

(3*S*,9*R*)-**1**

FIGURE S4

Populations; Conformers for P.F. (5%-1.5%) based on ΔE ΔE

CONFORMERS	ΔE	% pop
337	0.000000	12.5
44	0.042573	11.7
360	0.134554	10.0
23	0.307920	7.5
21	0.307982	7.5
26	0.328297	7.2
6	0.329300	7.2
79	0.329551	7.2
58	0.486803	5.5
18	0.559409	4.9
165	0.947522	2.5
124	0.981757	2.4
323	1.104899	1.9
196	1.230111	1.6
118	1.334256	1.3
250	1.403289	1.2
60	1.432820	1.1
19	1.568879	0.9
81	1.596844	0.8
227	1.663368	0.8
59	1.681802	0.7
262	1.879683	0.5
104	1.909842	0.5
269	1.967150	0.5
80	1.975928	0.4
154	1.978248	0.4
65	1.978310	0.4
2	2.169545	0.3
28	2.188669	0.3
127	2.400971	0.2

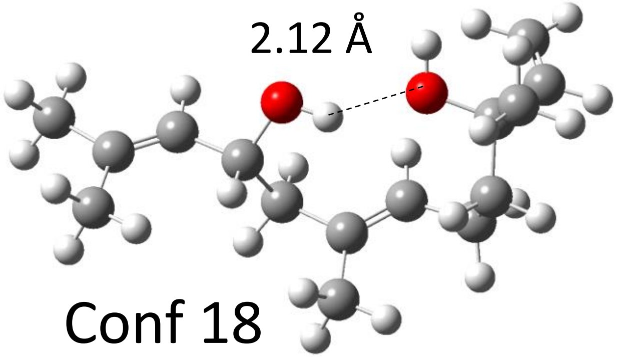

Conf 18

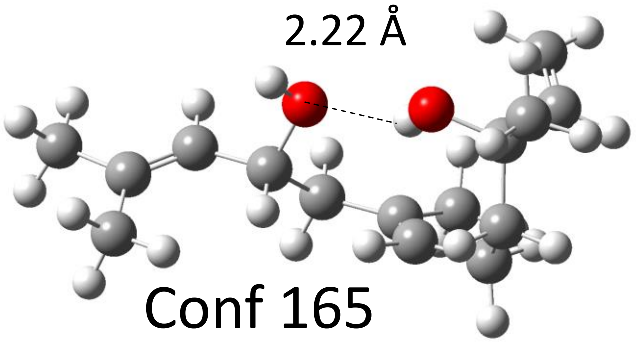

Conf 165

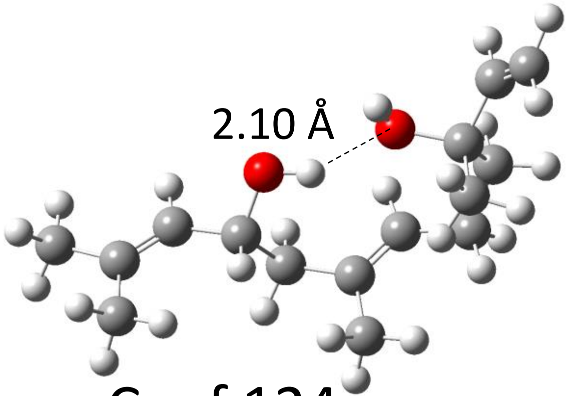

Conf 124

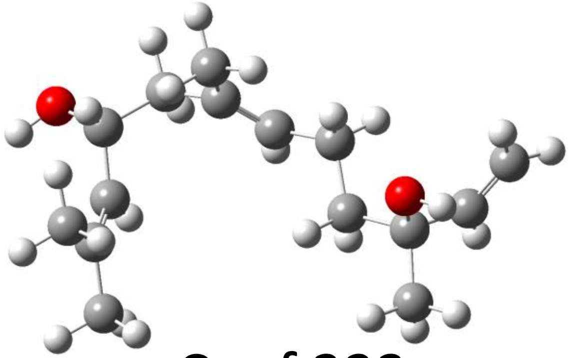
Conf 323

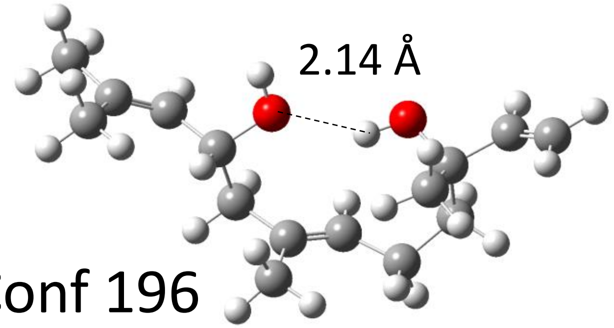

Conf 196

(3*S*,9*R*)-**1**

FIGURE S5

Populations; Conformers for (≥20%)-P.F.-based on ΔG

ΔG

CONFORMERS	ΔG	% pop
337	0.000000	28.1
360	0.030723	26.7
323	0.779361	7.5
104	1.010097	5.1
80	1.065273	4.7
79	1.106655	4.3
23	1.136124	4.1
6	1.350558	2.9
44	1.586937	1.9
18	1.622049	1.8
227	1.636470	1.8
59	1.673463	1.7
269	1.701051	1.6
58	1.771902	1.4
26	1.826451	1.3
165	2.101077	0.8
124	2.129292	0.8
21	2.215191	0.7
118	2.361909	0.5
60	2.415204	0.5
196	2.597034	0.4
28	2.650956	0.3
81	2.855358	0.2
262	2.980758	0.2
65	2.982012	0.2
154	2.985147	0.2
250	3.069792	0.2
19	3.377022	0.1
2	3.546312	0.1
127	3.853542	0.0

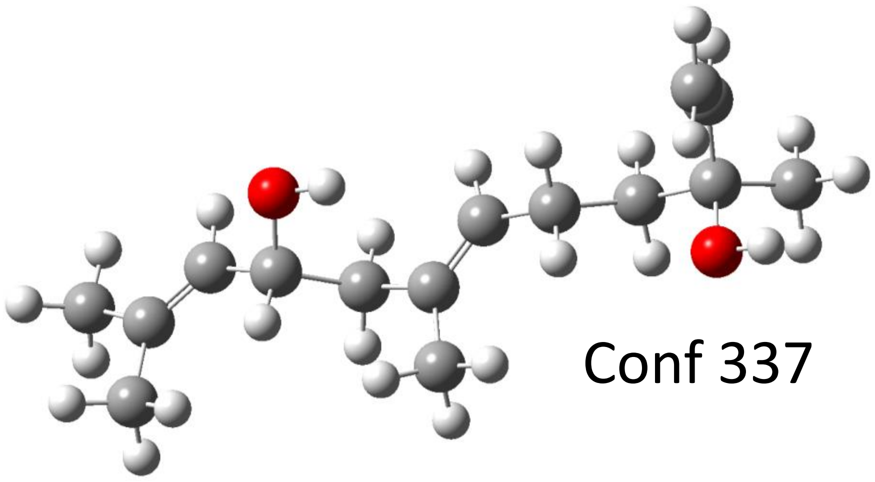

Conf 337

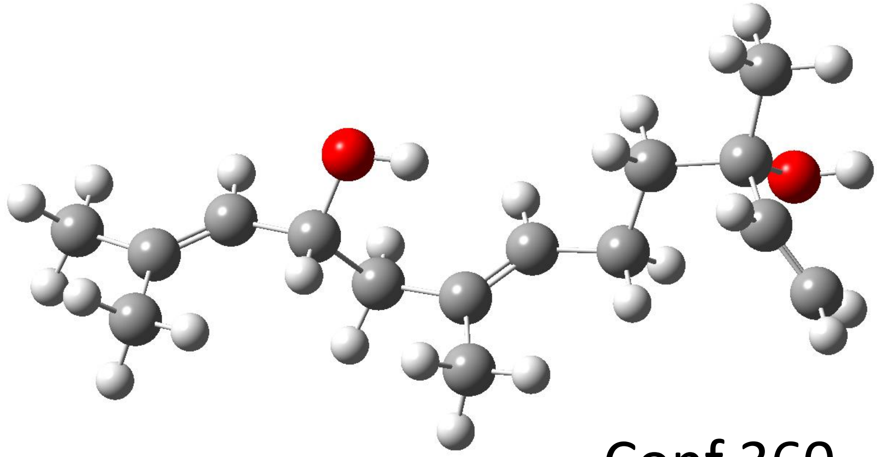

Conf 360

(3S,9R)-**1**

FIGURE S6

Populations; Conformers for $(9\% - 2\%)$P.F.-based on ΔG

ΔG

CONFORMERS	ΔG	% pop
337	0.000000	28.1
360	0.030723	26.7
323	0.779361	7.5
104	1.010097	5.1
80	1.065273	4.7
79	1.106655	4.3
23	1.136124	4.1
6	1.350558	2.9
44	1.586937	1.9
18	1.622049	1.8
227	1.636470	1.8
59	1.673463	1.7
269	1.701051	1.6
58	1.771902	1.4
26	1.826451	1.3
165	2.101077	0.8
124	2.129292	0.8
21	2.215191	0.7
118	2.361909	0.5
60	2.415204	0.5
196	2.597034	0.4
28	2.650956	0.3
81	2.855358	0.2
262	2.980758	0.2
65	2.982012	0.2
154	2.985147	0.2
250	3.069792	0.2
19	3.377022	0.1
2	3.546312	0.1
127	3.853542	0.0

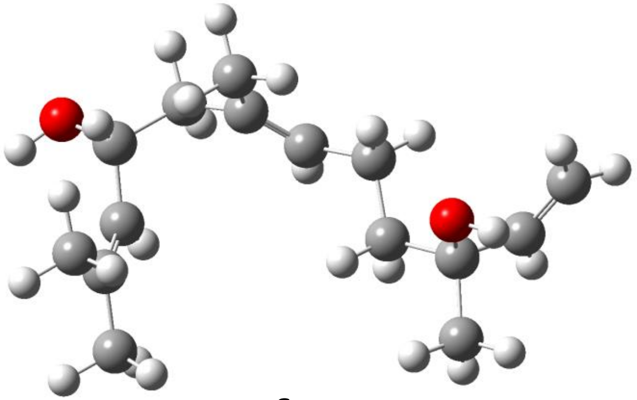
Conf 323

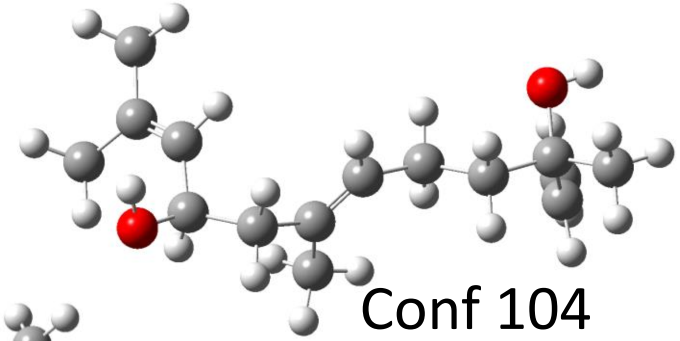
Conf 104

<1% in ΔE

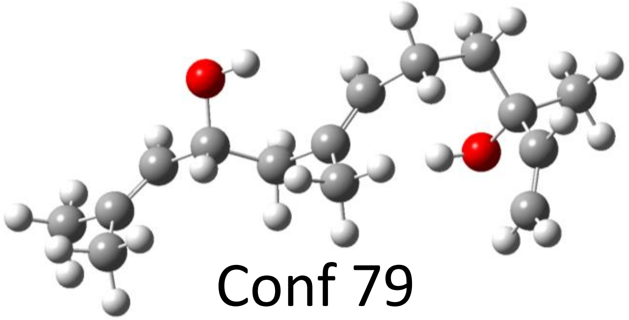
Conf 79

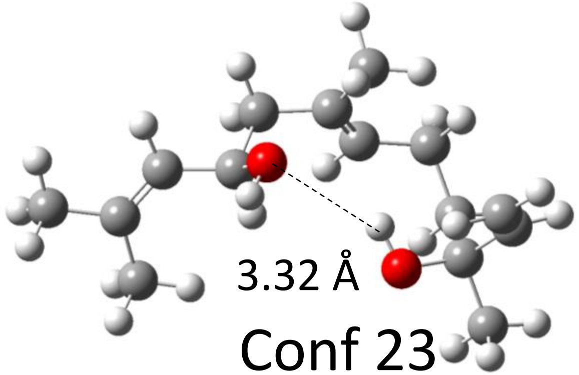

Conf 23

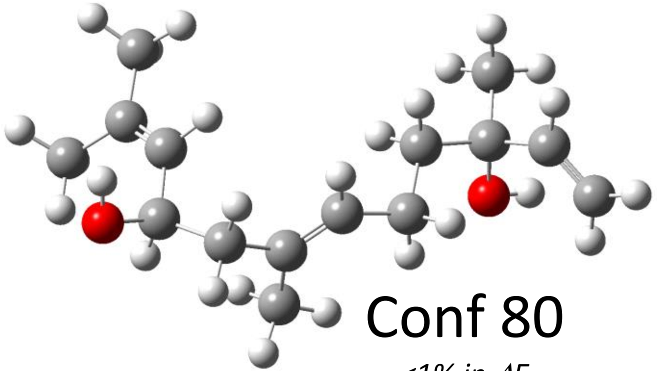
Conf 80

<1% in ΔE

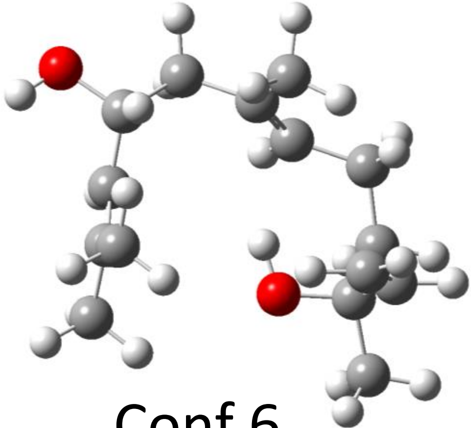
Conf 6

(3S,9R)-**1**

FIGURE S7

Populations; Conformers for (2% – 1%)P.F.-based on ΔG

ΔG

CONFORMERS	ΔG	% pop
337	0.000000	28.1
360	0.030723	26.7
323	0.779361	7.5
104	1.010097	5.1
80	1.065273	4.7
79	1.106655	4.3
23	1.136124	4.1
6	1.350558	2.9
44	1.586937	1.9
18	1.622049	1.8
227	1.636470	1.8
59	1.673463	1.7
269	1.701051	1.6
58	1.771902	1.4
26	1.826451	1.3
165	2.101077	0.8
124	2.129292	0.8
21	2.215191	0.7
118	2.361909	0.5
60	2.415204	0.5
196	2.597034	0.4
28	2.650956	0.3
81	2.855358	0.2
262	2.980758	0.2
65	2.982012	0.2
154	2.985147	0.2
250	3.069792	0.2
19	3.377022	0.1
2	3.546312	0.1
127	3.853542	0.0

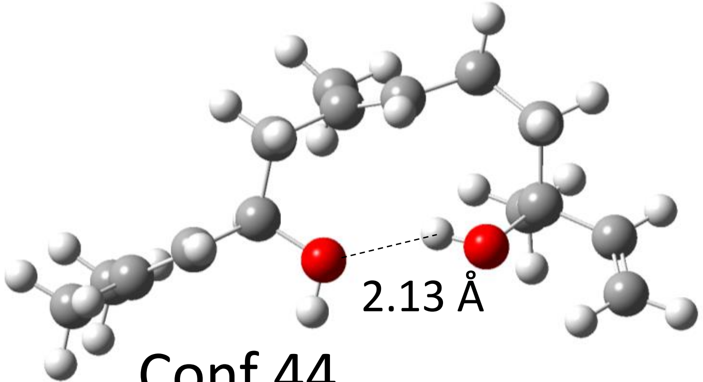

Conf 44

11.7% in ΔE

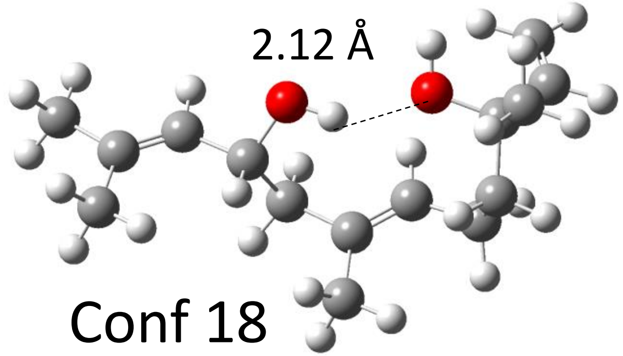

Conf 18

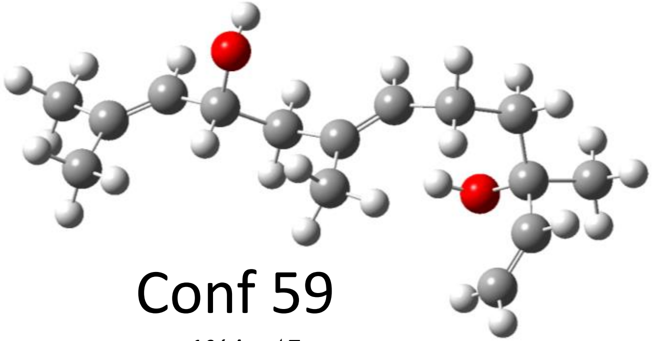

Conf 59

<1% in ΔE

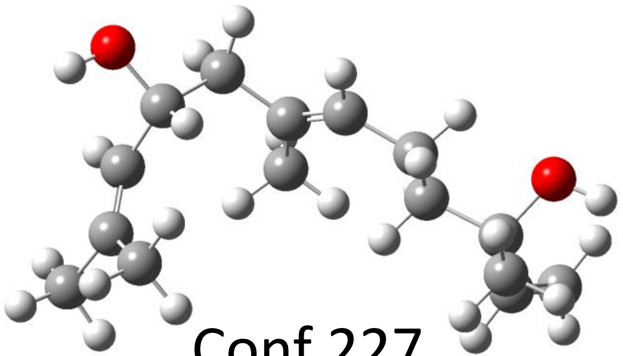

Conf 227

<1% in ΔE

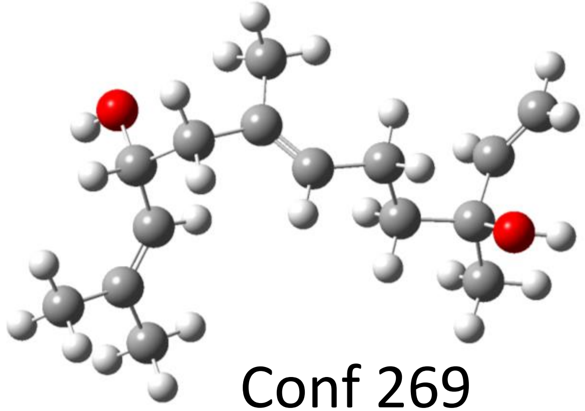

Conf 269

<1% in ΔE

(3*S*,9*R*)-**1**

TABLE S3

1: AC (3*S*,9*S*) Computed Poputation Factors

Conformational Search B3LYP/6-31G* on 264 conformers.
Re-optimized with frequency calculated at B3LYP/6-311++G(2d,p)

[$] Original numbering of the conformers from the MM conformational search has been maintained

ΔE

CONFORMERS	ΔE	% pop
16	0.000000	45.8
1	0.732775	13.3
4	0.742180	13.1
61	1.209358	6.0
38	1.271870	5.4
57	1.512324	3.6
90	1.643367	2.9
2	1.684373	2.7
153	1.800368	2.2
85	1.967526	1.7
24	2.009472	1.5
14	2.208921	1.1
48	2.655157	0.5
115	2.944643	0.3
240	4.159581	0.0

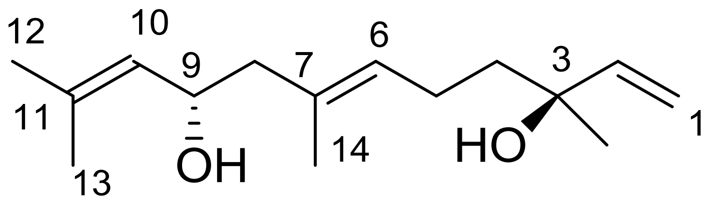

1, CL1, 9-Hydroxynerolidol

ΔG

CONFORMERS	ΔG	% pop
4	0.000000	73.4
1	0.732775	9.9
38	0.742180	5.3
57	1.209358	3.2
16	1.271870	1.5
61	1.512324	1.5
14	1.643367	1.4
2	1.684373	1.2
90	1.800368	0.8
153	1.967526	0.6
24	2.009472	0.6
85	2.208921	0.3
48	2.655157	0.2
115	2.944643	0.1
240	4.159581	0.1

(3*S*,9*S*)-**1**

FIGURE S8

Populations; Conformers for (≥ 20%)-P.F.-based on ΔE

ΔE

CONFORMERS	ΔE	% pop
16	0.000000	45.8
1	0.732775	13.3
4	0.742180	13.1
61	1.209358	6.0
38	1.271870	5.4
57	1.512324	3.6
90	1.643367	2.9
2	1.684373	2.7
153	1.800368	2.2
85	1.967526	1.7
24	2.009472	1.5
14	2.208921	1.1
48	2.655157	0.5
115	2.944643	0.3
240	4.159581	0.0

Conf 16

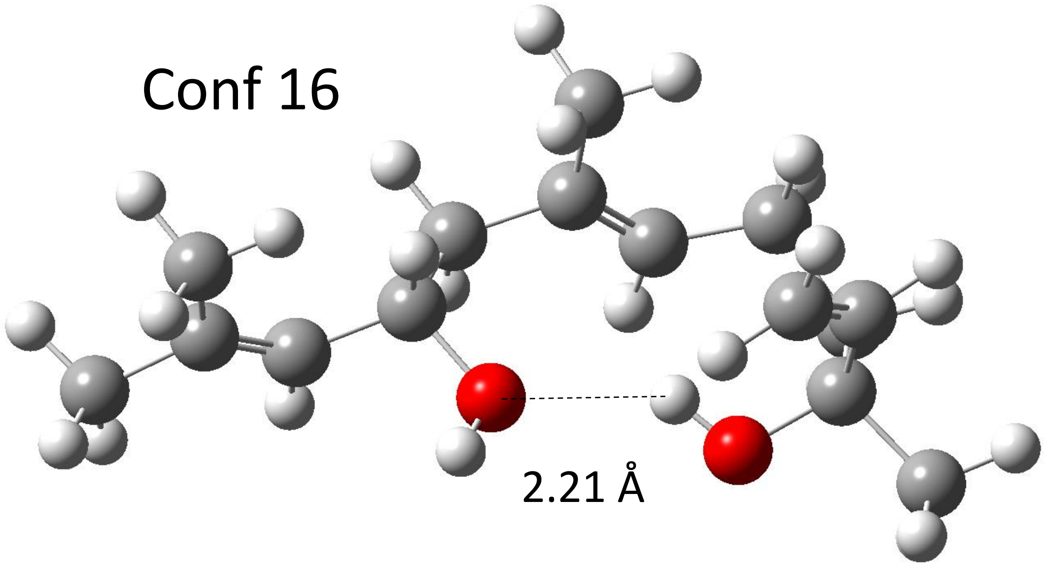

(3*S*,9*S*)-**1**

FIGURE S9

Populations; Conformers for (20%-10%)-P.F.-based on ΔE

ΔE

CONFORMERS	ΔE	% pop
16	0.000000	45.8
1	0.732775	13.3
4	0.742180	13.1
61	1.209358	6.0
38	1.271870	5.4
57	1.512324	3.6
90	1.643367	2.9
2	1.684373	2.7
153	1.800368	2.2
85	1.967526	1.7
24	2.009472	1.5
14	2.208921	1.1
48	2.655157	0.5
115	2.944643	0.3
240	4.159581	0.0

Conf 1

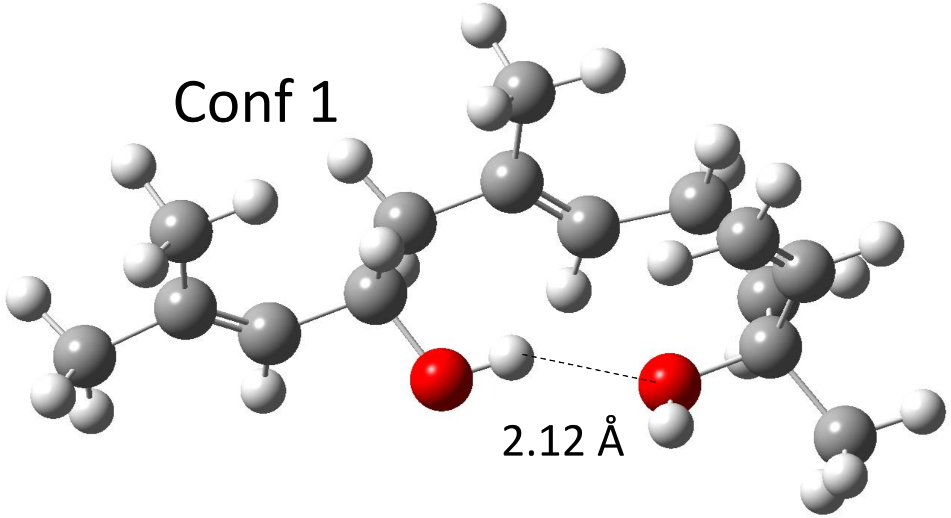

Conf 4

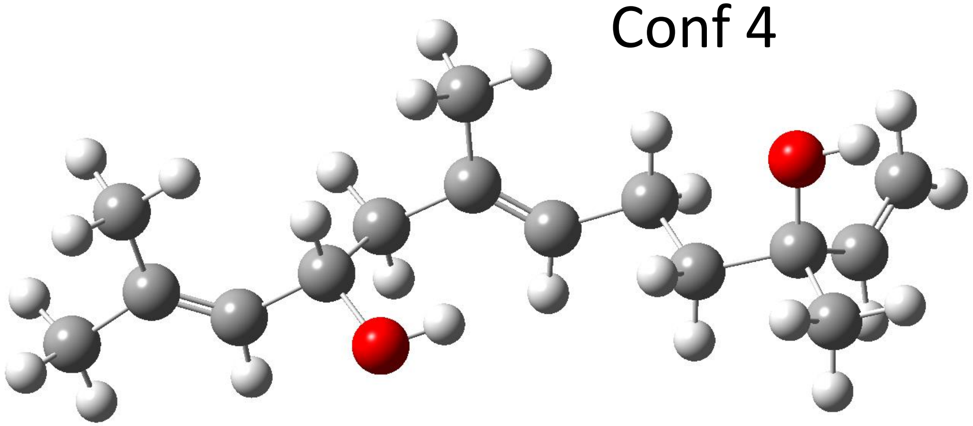

(3*S*,9*S*)-**1**

Populations; Conformers for P.F. (10%-3%) based on ΔE

ΔE

CONFORMERS	ΔE	% pop
16	0.000000	45.8
1	0.732775	13.3
4	0.742180	13.1
61	1.209358	6.0
38	1.271870	5.4
57	1.512324	3.6
90	1.643367	2.9
2	1.684373	2.7
153	1.800368	2.2
85	1.967526	1.7
24	2.009472	1.5
14	2.208921	1.1
48	2.655157	0.5
115	2.944643	0.3
240	4.159581	0.0

Conf 61

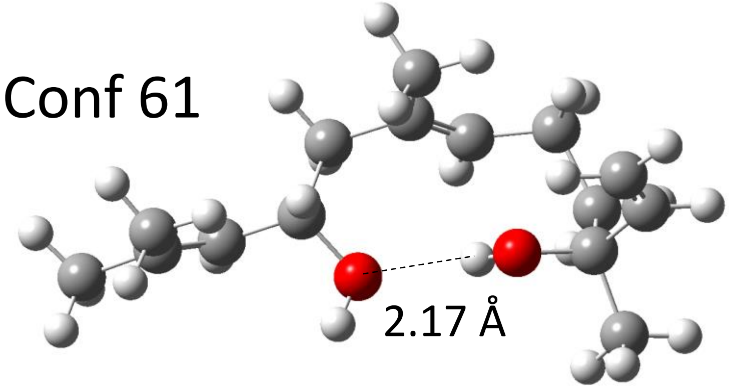

Conf 38

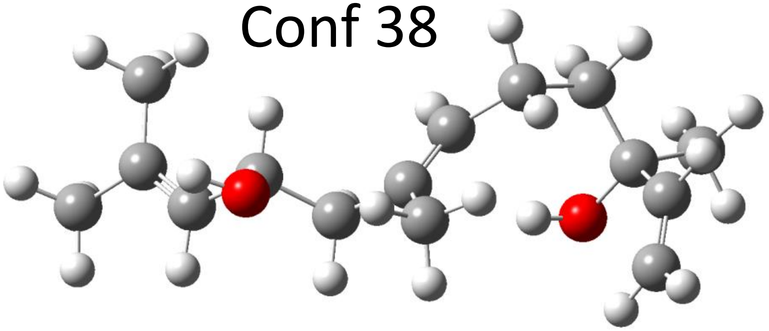

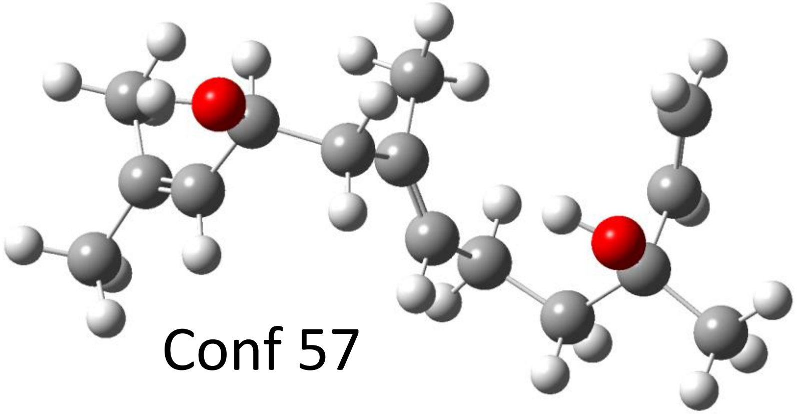

Conf 57

(3*S*,9*S*)-**1**

FIGURE S11

Populations; Conformers for P.F. based on ΔE (<3%)

ΔE

CONFORMERS	ΔE	% pop
16	0.000000	45.8
1	0.732775	13.3
4	0.742180	13.1
61	1.209358	6.0
38	1.271870	5.4
57	1.512324	3.6
90	1.643367	2.9
2	1.684373	2.7
153	1.800368	2.2
85	1.967526	1.7
24	2.009472	1.5
14	2.208921	1.1
48	2.655157	0.5
115	2.944643	0.3
240	4.159581	0.0

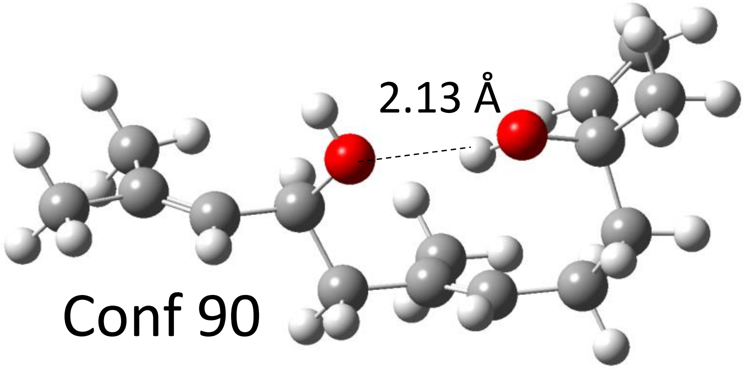

Conf 90

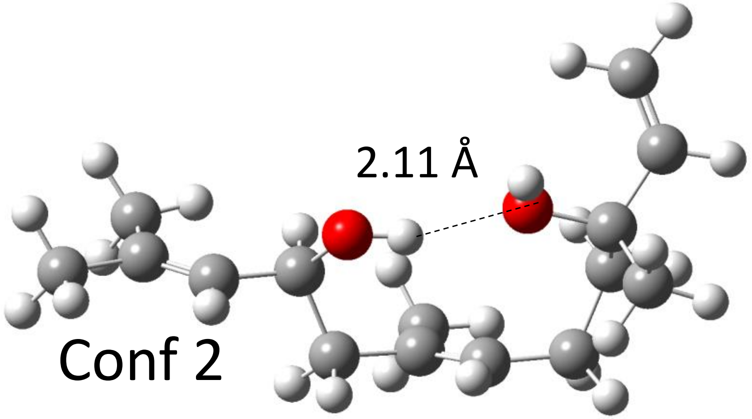

Conf 2

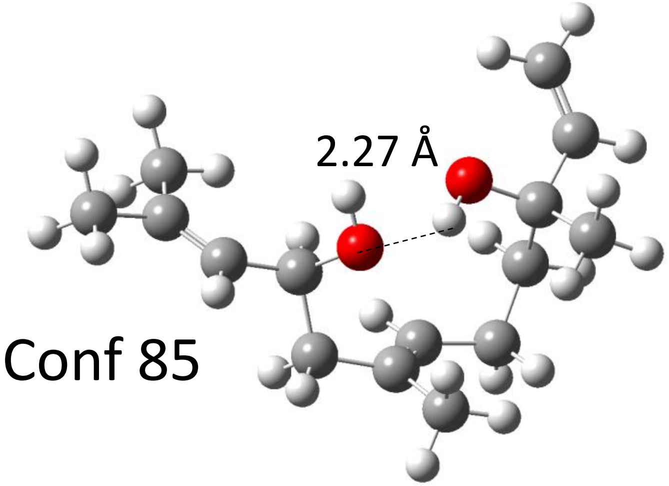

Conf 85

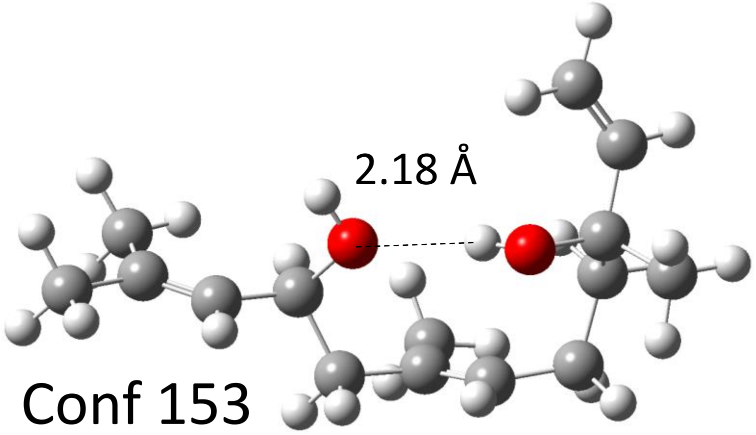

Conf 153

(3*S*,9*S*)-**1**

FIGURE S12

Populations; Conformers for (≥ 20%)-P.F.-based on ΔG

ΔG

CONFORMERS	ΔG	% pop
4	0.000000	73.4
1	0.732775	9.9
38	0.742180	5.3
57	1.209358	3.2
16	1.271870	1.5
61	1.512324	1.5
14	1.643367	1.4
2	1.684373	1.2
90	1.800368	0.8
153	1.967526	0.6
24	2.009472	0.6
85	2.208921	0.3
48	2.655157	0.2
115	2.944643	0.1
240	4.159581	0.1

Conf 4

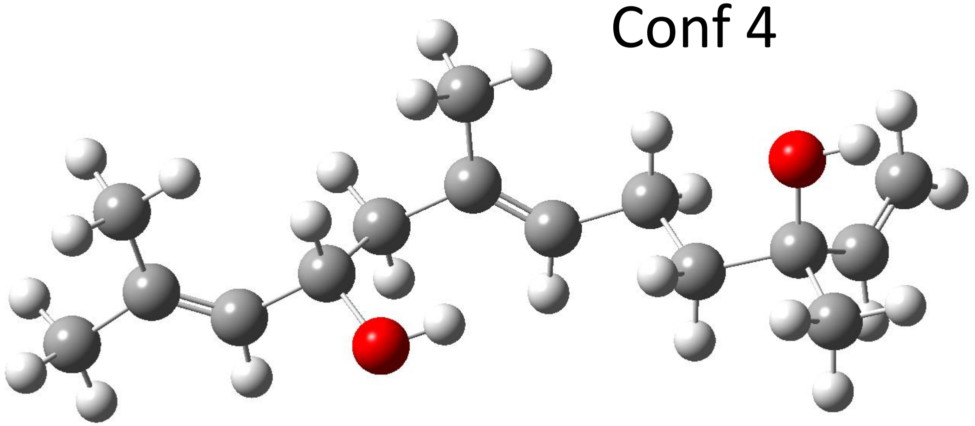

(3*S*,9*S*)-**1**

Populations; Conformers for (10%-3%) based on ΔG

ΔG

CONFORMERS	ΔG	% pop
4	0.000000	73.4
1	0.732775	9.9
38	0.742180	5.3
57	1.209358	3.2
16	1.271870	1.5
61	1.512324	1.5
14	1.643367	1.4
2	1.684373	1.2
90	1.800368	0.8
153	1.967526	0.6
24	2.009472	0.6
85	2.208921	0.3
48	2.655157	0.2
115	2.944643	0.1
240	4.159581	0.1

Conf 1

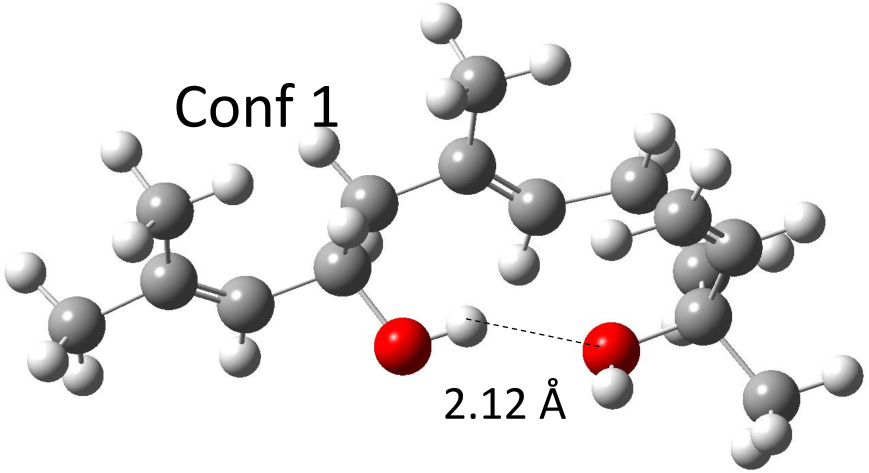

Conf 38

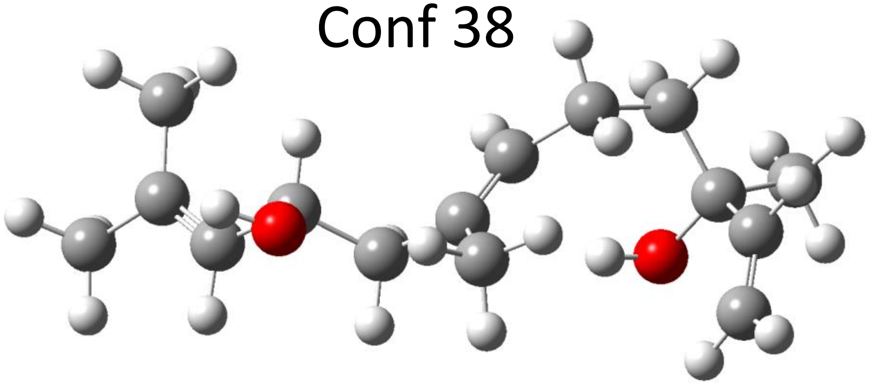

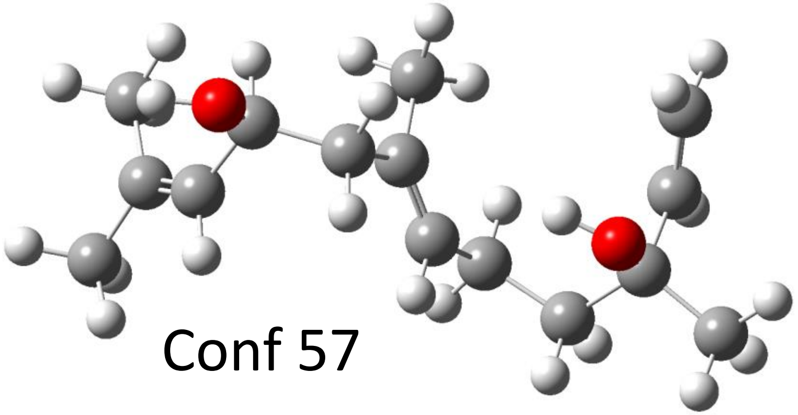

Conf 57

(3*S*,9*S*)-**1**

FIGURE S14

Populations; Conformers for (3%-1%) based on ΔG

ΔG

CONFORMERS	ΔG	% pop
4	0.000000	73.4
1	0.732775	9.9
38	0.742180	5.3
57	1.209358	3.2
16	1.271870	1.5
61	1.512324	1.5
14	1.643367	1.4
2	1.684373	1.2
90	1.800368	0.8
153	1.967526	0.6
24	2.009472	0.6
85	2.208921	0.3
48	2.655157	0.2
115	2.944643	0.1
240	4.159581	0.1

Conf 16

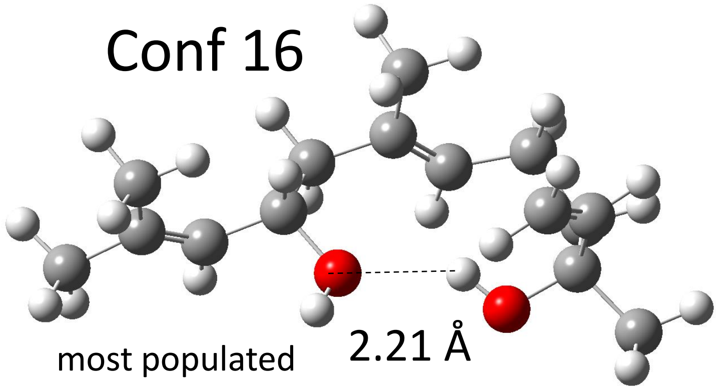

most populated conformer according to ΔE

Conf 61

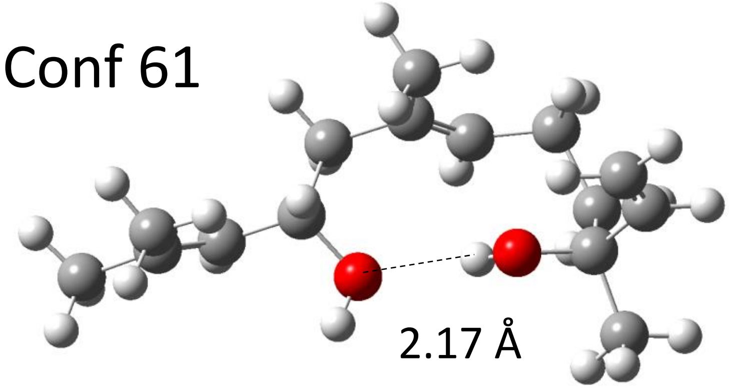

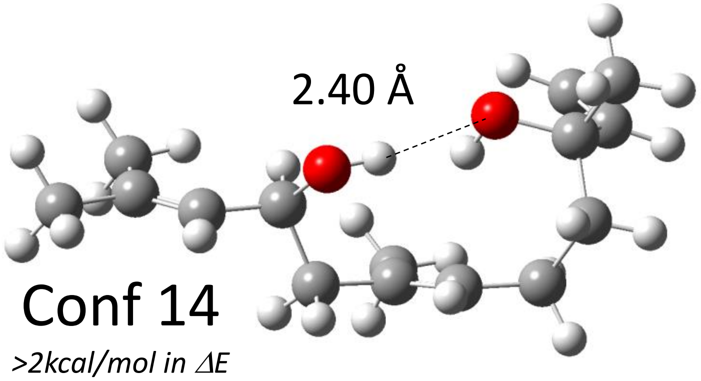

Conf 14

>2kcal/mol in ΔE

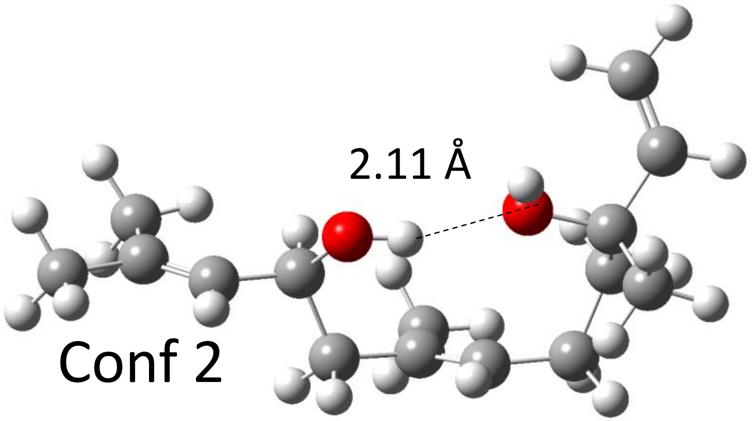

Conf 2

(3*S*,9*S*)-**1**
